# Predicting Overall Survival with Deep Learning from 18F-FDG PET-CT Images in Patients with Hepatocellular Carcinoma before Liver Transplantation

**DOI:** 10.3390/diagnostics13050981

**Published:** 2023-03-04

**Authors:** Yung-Chi Lai, Kuo-Chen Wu, Chao-Jen Chang, Yi-Jin Chen, Kuan-Pin Wang, Long-Bin Jeng, Chia-Hung Kao

**Affiliations:** 1 Department of Nuclear Medicine, Feng Yuan Hospital, Ministry of Health and Welfare, Taichung 420210, Taiwan; 2 Department of Nuclear Medicine, PET Center, China Medical University Hospital, Taichung 404327, Taiwan; 3 Graduate Institute of Biomedical Electronics and Bioinformatics, National Taiwan University, Taipei 106319, Taiwan; 4 Artificial Intelligence Center, China Medical University Hospital, Taichung 404327, Taiwan; 5 Department of Computer Science and Engineering, National Chung Hsing University, Taichung 402202, Taiwan; 6 Organ Transplantation Center, China Medical University Hospital, Taichung 404327, Taiwan; 7 Graduate Institute of Biomedical Sciences, College of Medicine, China Medical University, Taichung 404327, Taiwan; 8 Department of Bioinformatics and Medical Engineering, Asia University, Taichung 413305, Taiwan

**Keywords:** 18F-fluorodeoxyglucose (18F-FDG), positron emission tomography and computed tomography (PET-CT), hepatocellular carcinoma (HCC), liver transplantation (LT), deep learning

## Abstract

Positron emission tomography and computed tomography with 18F-fluorodeoxyglucose (18F-FDG PET-CT) were used to predict outcomes after liver transplantation in patients with hepatocellular carcinoma (HCC). However, few approaches for prediction based on 18F-FDG PET-CT images that leverage automatic liver segmentation and deep learning were proposed. This study evaluated the performance of deep learning from 18F-FDG PET-CT images to predict overall survival in HCC patients before liver transplantation (LT). We retrospectively included 304 patients with HCC who underwent 18F-FDG PET/CT before LT between January 2010 and December 2016. The hepatic areas of 273 of the patients were segmented by software, while the other 31 were delineated manually. We analyzed the predictive value of the deep learning model from both FDG PET/CT images and CT images alone. The results of the developed prognostic model were obtained by combining FDG PET-CT images and combining FDG CT images (0.807 AUC vs. 0.743 AUC). The model based on FDG PET-CT images achieved somewhat better sensitivity than the model based on CT images alone (0.571 SEN vs. 0.432 SEN). Automatic liver segmentation from 18F-FDG PET-CT images is feasible and can be utilized to train deep-learning models. The proposed predictive tool can effectively determine prognosis (i.e., overall survival) and, thereby, select an optimal candidate of LT for patients with HCC.

## 1. Introduction

Hepatocellular carcinoma (HCC) is a prevalent malignancy worldwide [1,2]. Liver cancer is Taiwan’s second-leading cause of cancer death [3]. Although surgical treatment results in the best long-term survival, most patients with HCC are not eligible due to either an underlying liver dysfunction or the extent of the tumor. Liver transplantation (LT) is a curative treatment for patients with hepatocellular carcinoma (HCC) [4,5,6,7]. LT is the only treatment that offers the possibility of eliminating the tumor and the underlying cirrhosis through the complete extirpation of both. LT outcomes for early HCC are somewhat encouraging; however, because of the limited supply of organs for transplantation, appropriate candidates must be selected to ensure successful results. In Asia, living donor LT (LDLT) has emerged as the leading solution to organ shortage when treating HCC.

All patients being considered for transplantation should undergo an evaluation for extra-hepatic malignancies. The imaging modalities include computed tomography (CT) of the chest or magnetic resonance imaging (MRI) of the abdomen and pelvis [8]. A bone scan was previously required, but this was changed in December 2012. Positron emission tomography using 18F-fluorodeoxyglucose (18F-FDG-PET) is a noninvasive functional technique that, in recent times, became standard in oncology [9,10,11]. Biologically, tumor 18F-FDG avidity by positron emission tomography (PET) can be a quantitative surrogate for tumor glucose metabolism, which is associated with tumor aggressiveness (e.g., tumor size or the presence of microvascular invasion) [12,13]. Additionally, 18F-FDG-PET was reported to be effective in identifying extra-hepatic metastases and ruling out recurrent HCC [14,15,16,17]. Some studies also determined that 18F-FDG-PET is a potent predictor of treatment outcomes in patients with HCC after undergoing hepatectomy, LT, radiofrequency ablation, and transarterial chemoembolization [10,11,18,19,20,21,22]. Hou et.al. recently proposed an integrative histology-genomic analysis to predict overall survival in hepatocellular carcinoma patients using deep learning [23,24]. Other established predictors for HCC recurrence include various biological markers related to tumor aggressiveness, such as tumor size, number, grade, stage, and microvascular invasion (mVI), as reported in several studies [25,26,27]. Therefore, evaluating the risk of HCC recurrence using these pretransplant biological markers is essential.

Thus, we hypothesized that findings from 18F-FDG-PET can be used to predict the long-term outcomes of LT for cancer therapy among patients with HCC. It is well known that the disease stage is closely related to prognosis in cancer patients. Resectable patients with early HCC are increasingly being considered for transplantation because of the potential for better disease-free survival, though this approach is limited by organ availability. Expanded transplantation criteria and downstaging to achieve transplant eligibility are now widely accepted. Pretransplant evaluation is essential because it helps the transplant team better understand the transplant candidacy. The decision to list a patient for transplantation is a risk-benefit analysis in which the inherent risks of surgery, recurrent disease, and long-term immunosuppression must be weighed against the potential benefits of transplantation. Early HCC recurrence portends the worst prognosis [28,29,30,31]. Patients with HCC recurrence within two years after liver transplantation had the highest mortality risk [32]. According to a systematic review that included 1021 cases of post-transplant HCC recurrence, the median post-recurrence survival was 13 months (0.1–112.5 months) [33]. Therefore, accurate estimates of recurrent disease and survival are of significant importance for patients and oncologists making personalized and patient-centered decisions in the era of precision medicine.

Medical imaging has developed rapidly, and radiomics has attracted increased attention with increases in the scale of data. Radiomic approaches utilize high-throughput calculations to extract patterns and quantitative features from standard medical images, such as tomographic images (i.e., computed tomography [CT], magnetic resonance imaging, and PET) that can be used for diagnosis, prognosis, and predicting treatment response. It involves using advanced algorithms and machine learning techniques to analyze the images and extract quantitative data. This data can then be used to classify tumors, predict survival rates, and identify patients who are more likely to respond to specific treatments. Radiomics has the potential to revolutionize the way that medical images are used in healthcare and could lead to more personalized and accurate diagnoses and treatments. In addition, radiomics is commonly used in oncology. Quantitative analyses of image intensity, texture, or shape minimize the subjectivity involved in image interpretation [34,35,36]. Additionally, artificial intelligence (AI) has yielded remarkable results in medical image diagnosis. Several state-of-the-art AI models, such as Visual Geometry Group and ResNet, are widely used in nuclear medical imaging [37].

Our present study included patients diagnosed with HCC who received LT. Given the diversity of HCC sizes, shapes, and locations, using the 18F-FDG-PET or CT solely is quite limiting. The database that we used included 18F-FDG-PET-CT images of these patients. We introduced automatic liver segmentation from the CT component of 18F-FDG-PET-CT images and then used the subsequent input to train a three-dimensional (3D) deep residual convolutional neural network that predicted the patient’s overall survival and intra-hepatic recurrence. We evaluated the model’s performance using 18F-FDG-PET-CT images.

In this study, we introduce the methods of extracting the images for data input (i.e., automatically extracting the hepatic areas), followed by the development of the deep learning model using both the PET/CT and CT images. Then, we evaluate the predictive performance of the proposed model. Finally, the possible applications and clinical significance are addressed.

## 2. Materials and Methods

### 2.1. Data Source

A total of 273 patients with hepatocellular carcinoma who had undergone an FDG-PET-CT scan followed by living donor LT at any time between January 2010 and December 2016 at China Medical University Hospital were enrolled in our retrospective study. This study was approved by the Institutional Review Board of our hospital (DMR99-IRB-010-[CR-13]).

### 2.2. Study Participants

**Figure A1:**
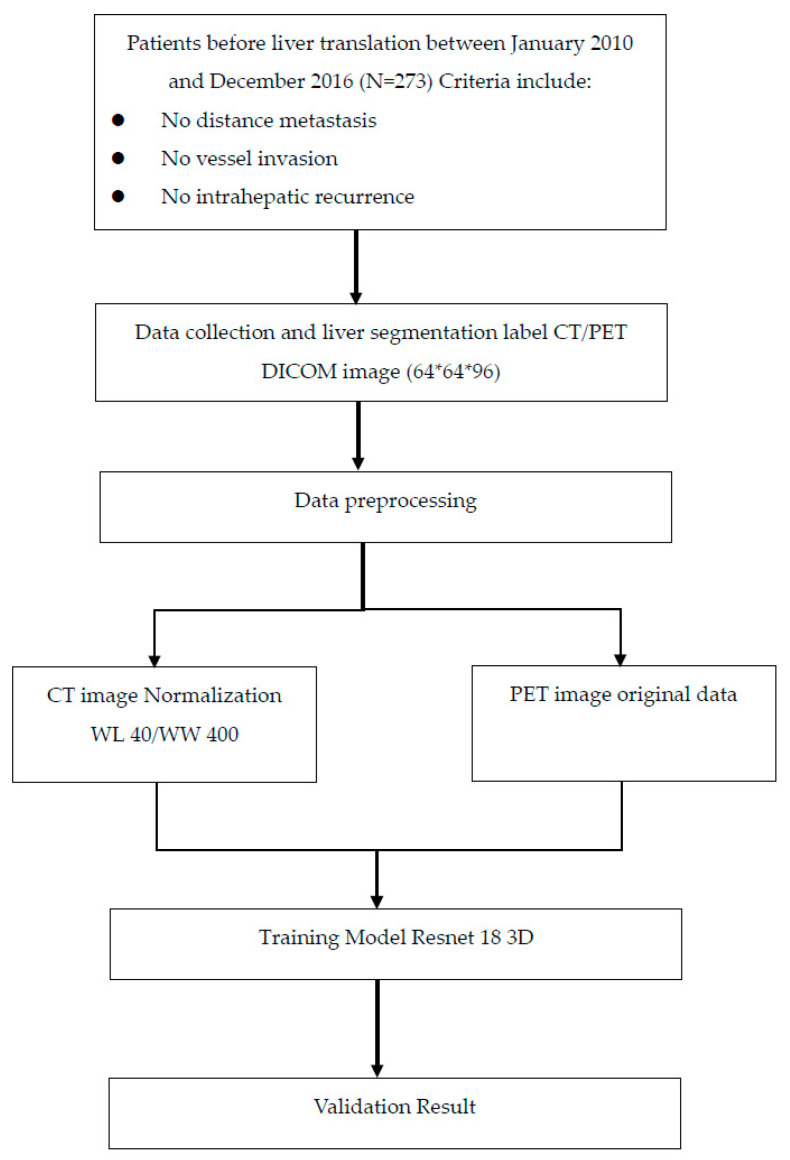
Flowchart.

The study retrospectively analyzed 273 images of patients diagnosed with hepatocellular carcinoma (HCC) who underwent 18F-FDG-PET-CT scans before undergoing liver transplantation at China Medical University Hospital between 2010 and 2016 (Table 1). The patients were instructed to fast for at least four hours before the scans, which were performed using a PET-CT scanner (Discovery STE, GE Medical Systems). Whole-body images were acquired approximately 45 min after the intravenous injection of 370 MBq of FDG, and delayed images were obtained about 70 min after the injection. Only the delayed images were used for further analysis [38,39,40], including automatic liver segmentation and input into deep-learning models. The images were reconstructed onto a 512 × 512 matrix with a section thickness of 3.75 mm and converted into 511-keV equivalent attenuation factors for attenuation correction. The maximum SUV max of hepatic tumors was measured for both early and delayed images (Figure A1).

### 2.3. Image Preprocessing

The 18F-FDG-PET-CT images were preprocessed before the ResNet-18 models were constructed. The process was roughly divided into the following steps.

In this study, we utilized the CT imaging technique to define the chest region of each patient. The CT images were viewed using the mediastinal window setting, with a window level of 40 and a window width of 400. This allowed for clear visualization of the mediastinal structures and facilitated the accurate identification of the chest region.

**Figure A2:**
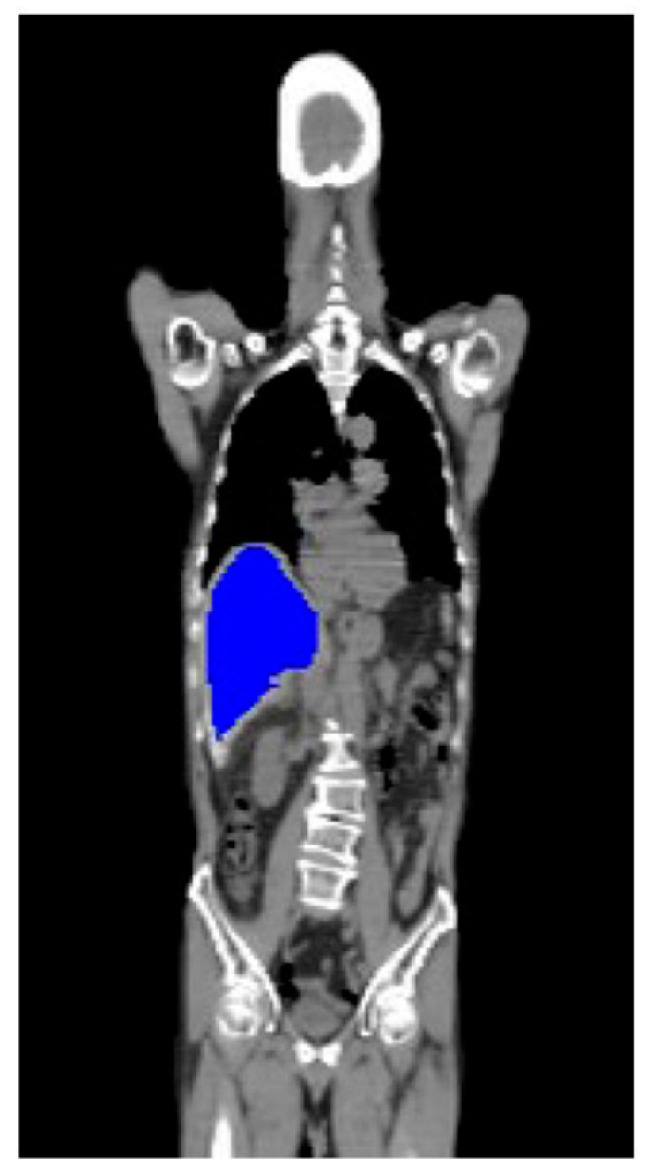
The liver 3D range is marked for training.

Automatic liver segmentation from 18F-FDG-PET-CT images is a highly crucial step in image preprocessing. To segment the hepatic areas, we leveraged semiautomated organ-contouring software to delineate the contour of the liver on the Digital Imaging and Communications in Medicine (DICOM) images. The tool is usually used in routine clinical radiotherapy planning to help radiologists confirm the location of cancer at which the radiation is aimed. We omitted the data outside the hepatic areas during model training and only adopted the CT and PET information within the liver areas (Figure A2).

In terms of data, the original size of the CT data was 512 × 512 pixels. The number of images and PET data points was 128 × 128 the number of images. The CT slices were symmetrically resized to a size of 128 × 128 pixels before model training, the same width as the PET images. We employed an image cropping technique in this study to obtain a standardized region of interest (VOI) from the marked CT images. The center point of the marked photos was determined as the starting point, and the part was then extended symmetrically in both left and right directions until the total area of the ROI was 64 × 64 pixels. This approach ensured that the ROI captured the relevant structures while maintaining a consistent size across all images. We then extended above and below the starting point until the thickness reached 96 image slices (the “solid rectangular extent of pixels”). Therefore, the maximum width of the grabbing range of the liver did not exceed 64 pixels and the height did not exceed 96 slices of images.

### 2.4. Deep Learning Model

**Figure A3:**
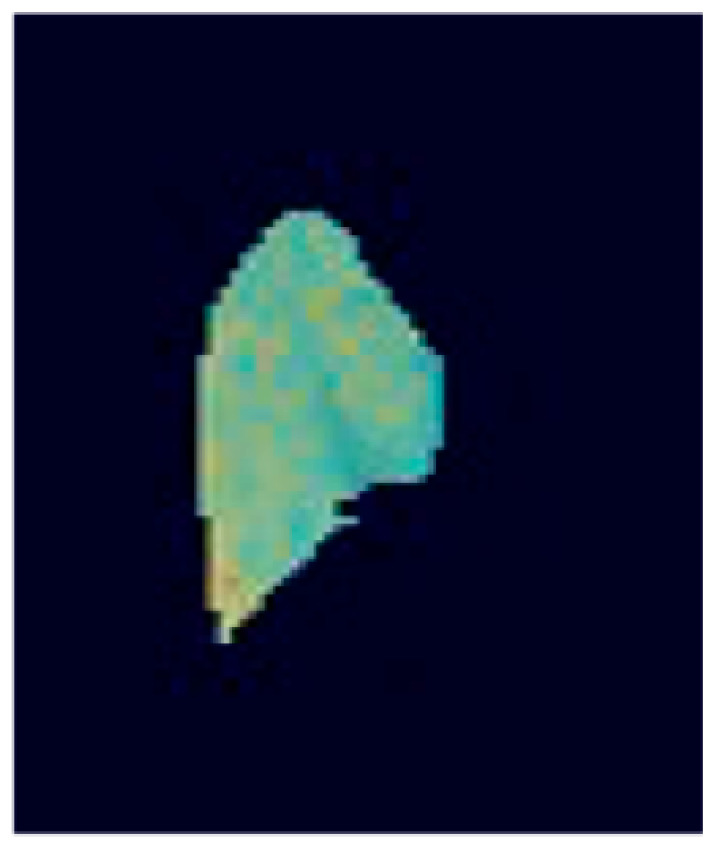
Only the CT and PET information of 64 × 64 × 96 pixels within the liver is retained.

We used the ResNet-18 3D model with a relatively small number of layers. Prior to model training, the CT and PET 3D images were cropped to 3D images of a size of 64 × 64 × 96 pixels (Figure A3). Because the image size was small, a low-parameter model could be used for training. In our proposed CT and PET image input process, two ResNet-18 models were combined by adding a connecting layer, which incorporated an independent dense layer at each end of the two models. The CT and PET models had their own independent convolution parameters. Additionally, only CT and PET images were used as data input in training. We compared the differences in training results between the two models.

### 2.5. Statistics and Assessment Methods

Statistical analyses were performed using SPSS version 26 (SPSS, NY, USA). The data were expressed in frequency (*n*), percentage (%), and mean ± standard deviation for normally distributed continuous variables. Categorical variables were compared using the χ^2^ test or Fisher’s exact test. We used Student’s *t*-test to compare continuous variables between groups, as appropriate. Logistic regression was used to identify significant variables for each group. Significance was indicated if *p* < 0.05 in a two-sided test.

Various metrics were used to evaluate the classification model’s performance on the test data. These validation metrics included accuracy (Equation (1)), sensitivity (Equation (2)), specificity (Equation (3)), and the area under the receiver operating characteristic curve. In a binary classification problem, predictions can be classified as true positive (TP), true negative (TN), false positive (FP), or false negative (FN). For the prediction of alive status at one year after LT, a TP meant that a patient who was predicted to die within one year died within one year after LT. FP meant that a patient who was predicted to die within one year survived for more than one year. TN meant that a patient who was predicted to die within one year died within one year. Finally, FN meant that a patient who was predicted to die within one year died within one year.

(1) $$\mathrm{Accuracy}\;=\frac{\mathrm{TP}\;+\;\mathrm{TN}}{\begin{array}{c} \left(\mathrm{TP}\;+\;\mathrm{FP}\;+\;\mathrm{TN}\;+\;\mathrm{FN}\right) \end{array}}$$

(2) $$\mathrm{Sensitivity}\;=\frac{\mathrm{TP}}{\mathrm{TP}\;+\;\mathrm{FN}}$$

(3) $$\mathrm{Specificity}\;=\frac{\mathrm{TN}}{\mathrm{FP}\;+\;\mathrm{TN}}$$

## 3. Results

Our study used two types of data as input (i.e., FDG-PET/CT and CT images alone) to train deep learning algorithms that classified liver recipients who died within or survived for more than one year after LT.

**Figure 1:**
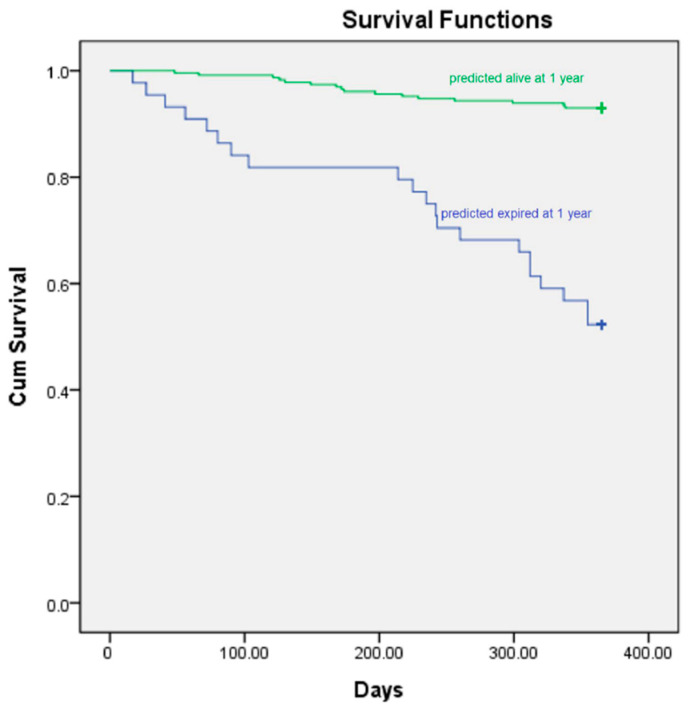
Prognostic significance of PET-CT base prediction results.

To investigate prediction performance, a survival analysis was performed (Figure 1). We evaluated the model performance for two groups of images. First, the model that processed both FDG-PET and CT images had a relatively better sensitivity (0.571 vs. 0.432 SEN) than the one that only processed CT images.

We defined SEN as the number of patients who were correctly predicted to die within one year divided by the total number of patients that died within one year; thus, sensitivity was an indicator of reliability. The prediction performances of the proposed models are detailed in the tables in the Appendix A.

All the images in the training data set underwent automatic hepatic segmentation using the aforementioned semiautomated-organ-contouring software. Of these, 37 patients (14%) died within one year after LT, and the other 236 patients (86%) survived for more than one year. However, the validation cohort comprised images that could not be segmented by software successfully; thus, manual delineation was conducted instead.

## 4. Discussion

Several studies demonstrated that 18F FDG PET-CT can predict outcomes in HCC patients after surgical resection. In addition, much research has demonstrated the usefulness of machine learning in the evaluation of post-therapeutic prognosis in patients with HCC [20,41,42,43,44,45,46,47,48,49,50,51,52,53,54]. However, no article took advantage of deep learning from 18F FDG PET-CT images to evaluate outcomes of HCC patients undergoing living donor liver transplantation.

The uptake of F-18 fluorodeoxyglucose (FDG) in hepatocellular carcinoma (HCC) is related to tumor biology and can serve as a predictor of tumor recurrence after liver transplantation (LT). Studies showed that positive FDG uptake significantly predicted worse recurrence-free survival (RFS) in patients with HCC who had undergone LT. For example, Hsu et al. [55] reported that the 3-year RFS of FDG-negative patients was significantly better than that of FDG-positive patients (93% vs. 35%). Similarly, Yang et al. found that the 2-year RFS of FDG-negative patients was considerably better than that of FDG-positive patients (85.1% vs. 46.1%). Additionally, FDG-positive status was found to be an independent predictor of early HCC recurrence (within six months) in a study by Lee et al. [56], which analyzed 191 patients who underwent FDG-PET scans and subsequent living donor liver transplantation (LDLT) for HCC. Furthermore, Hsu et al. demonstrated that the degree of FDG uptake was associated with HCC recurrence and contributed to the risk of HCC recurrence after LDLT. Therefore, it can be concluded that FDG-PET can predict HCC recurrence after living donor liver transplantation.

**Figure A4:**
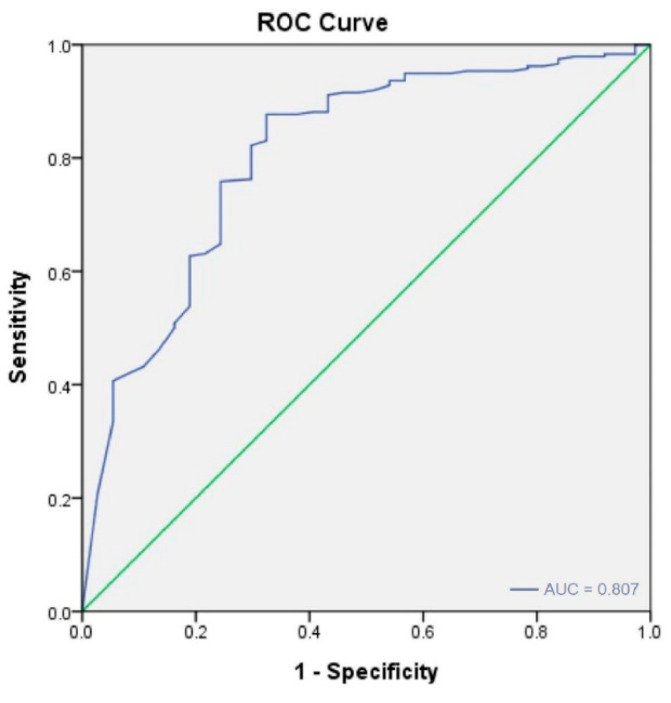
The area under the curve of 0.807 for the PET-CT base.

Researchers offered benchmarks for classifying area under the curve (AUC) results, suggesting that the values ≥0.9, ≥0.80, ≥0.70, ≥0.60, and <0.60 indicate excellent, good, fair, poor, and unacceptable predictive performance, respectively. However, these are likely to be appropriate for engineering and some applications in biomedicine but less so for mental health diagnoses [57,58,59]. Our deep learning models gave fair to good results with respect to the AUC values. Please refer to Figure A4 in the Appendix A for details of the ROC.

Deep learning technology has changed rapidly. For example, computing resources are becoming more advanced, and data sets more extensive. Neural networks have typically been applied to two-dimensional data; however, our study proposed a 3D neural network model, where the liver’s FDG-PET/CT data were directly input into the model for training. Overfitting is a problem in deep learning that becomes more serious with the more superimposed neural network layers there are. Therefore, various architectures must be developed to determine the best solution. At times, the use of a small number of layers can yield superior predictive accuracy. Therefore, we selected the shallower ResNet-18, professional computing server DGX-2, for our study. The difficulty in applying deep learning lies in quantifying a real-world problem and making it amenable to being solved by machine learning. Undoubtedly, deep learning will be more widely used in the future.

The shortage of donor organs is a challenging global problem. Thus, organ shortage is becoming an increasing problem worldwide, limiting the applicability of liver transplantation. Organ shortage may deprive many patients of a new and better quality of life and may cause a substantial increase in the cost of alternative medical care. In addition, potential recipients are becoming more ill, thereby increasing the risk of losing the graft during transplantation or in the initial postoperative period after liver transplantation. Thus, selecting the most appropriate candidates, that is, who is likely to survive longer after transplantation, is of great importance currently. Unnecessary surgical treatment, including liver resection and liver transplantation, can result in potential morbidity and mortality. However, in current clinical practice, few prediction tools can be confidently used in HCC patients. In the present study, we developed a deep-learning algorithm with a view to predicting survival outcomes in HCC patients following liver transplantation. It is exciting that the performance of the proposed deep-learning model was satisfactory. By using this versatile automatic technique, surgeons and patients can gain a general understanding of the outcomes before considering liver transplantation as the primary therapeutic option. Primary care physicians can fulfill the “do no harm” principle in medicine and benefit the patients most.

In the future, we hope to develop a deep-learning prognostic model combining baseline clinical characteristics and FDG PET/CT. The baseline clinical parameters may include the initial staging of HCC by various staging systems and biological markers such as alpha-fetal protein level. Serum alpha-fetoprotein (AFP) level is associated with clinical outcomes in patients with HCC undergoing LDLT [60]. AFP is an attractive prognostic maker that has been studied extensively in HCC. AFP may be a surrogate for vascular invasion and a predictor of HCC recurrence. Numerous studies demonstrated the predictive utility of pretransplant AFP level, although no validated threshold that can be applied across patient groups is available. Takada et al. [61] proposed new selection criteria for living donor liver transplantation based on the Milan criteria: AFP < 115 ng/mL and [18F] FDG-PET avidity. In addition to tumor size, AFP is a surrogate marker for tumor microvascular invasion; such invasion is also a known predictor of poor outcomes [62]. We hope to use data on the AFP level to train the deep learning models because adding such data may increase the prediction performance.

The limitations of this study were as follows. First, the method employed herein was to train the network on those cases in which the automatic segmentation method could be employed and test this network on the cases in which the automatic segmentation did not work, and segmentation had to be performed manually. This introduced potential bias into the results. In our future research, we hope to randomly split a population of subjects into training and test populations when using artificial neural networks. Second, this study had a limited number of participants, and an extensive multicenter study with external validation is required for further verification of the results.

The significant findings of our study were as follows. First, deep learning can be used to predict the outcomes of patients with HCC following liver transplantation from FDG-PET-CT images. Second, the hepatic area can be automatically segmented with software for most cases. The segmented hepatic images can effectively serve as input for deep learning analysis. Most hepatic images could be extracted automatically through software in the absence of any manual delineation. Third, combining both FDG-PET and CT modalities as data input gave moderately better one-year survival outcome prediction results than the model derived solely from the CT images. Although our study only comprised 306 patients, the AI algorithm generally produced accurate and reliable results.

Better survival outcomes in patients with HCC are attributed to excellent surgical results and optimal patient selection. By using deep learning-based models, the present study attempted to predict the outcome improvement before the patient received LT and facilitated the selection of optimal candidates. The database included FDG-PET-CT images of the liver, and by analyzing them with the proposed deep learning algorithm, we hope our robust tool will help to predict outcomes in patients with HCC before LT. Moreover, an online platform based on this versatile predictive tool can be established to provide better medical planning and optimize decision-making for medical physicians and patients.

## 5. Conclusions

Our retrospective study indicated that an automated 3D ResNet-18 convolutional neural network with FDG-PET-CT has promise for predicting clinical outcomes in patients with HCC undergoing LDLT. A significant strength of the proposed deep learning algorithm is that it can automatically segment the hepatic area from the CT component of FDG-PET-CT without any time-consuming manual delineation. Furthermore, the predictive performance was quite satisfactory when FDG-PET and CT images served as input data for the ResNet-18 model. However, in this study, a relatively small sample size was used, which may have limited the generalizability of our findings. Therefore, it is essential to note that our results should be interpreted with caution and validated in larger, multicenter studies. This will help establish our findings’ external validity and ensure that they can be applied to a broader population. Moreover, this will increase the study’s statistical power, allowing for more robust conclusions.

## Figures and Tables

**Table 1:** Patient Characteristics.

Total	
*n* = 273	
Age (years, mean ± SD)	55.773 ± 8.138
Gender	
Male	212 (77.7)
Female	61 (22.3)
BCLC Classification	
0	1 (0.4)
A	119 (43.6)
B	89 (32.6)
C	40 (14.7)
D	24 (8.8)
Milan criteria	
within	127 (46.5)
beyond	146 (53.5)
UCSF criteria	
within	147 (53.8)
beyond	126 (46.2)
CLIP Score	
0	61 (22.3)
1	105 (38.5)
2	48 (17.6)
3	37 (13.6)
4	17 (6.2)
>4	5 (1.8)
Child–Pugh Classification	
Stage A	167 (61.2)
Stage B	81 (29.7)
Stage C	25 (9.2)
Okuda staging system	
Ⅰ	162 (59.3)
Ⅱ	91 (33.3)
Ⅲ	20 (7.3)
MELD Score	
<10	134 (49.1)
10–19	96 (35.2)
20–29	35 (12.8)
30–39	6 (2.2)
>39	2 (0.7)
Pretransplant AFP, ng/mL	
<20	139 (50.9)
20–200	74 (27.1)
>200	60 (22.0)

BCLC: Barcelona Clinic Liver Cancer; UCSF: University of California San Francisco; CLIP: Cancer of the Liver Italian Program; MELD: Median model for end-stage liver disease; AFP: alpha-fetoprotein; SD: standard deviation.

## Data Availability

Not applicable.

## Appendix A

**Table A1:** Comparison of prediction performances between the proposed models.

Methods	Training Set	Multiple Endpoints							
	Fold	AUC	TP	TN	FP	FN	Specificity	Sensitivity	Accuracy
PET + CT	Fold 1	0.817	45	5	2	3	0.93750	0.71429	0.90909
	Fold 2	0.707	42	4	4	5	0.89362	0.50000	0.83636
	Fold 3	0.851	45	4	4	2	0.95745	0.50000	0.89091
	Fold 4	0.915	42	5	2	5	0.89362	0.71429	0.87037
	Fold 5	0.657	40	3	4	7	0.85106	0.42857	0.79630
	mean	0.789	214	21	16	22	0.90665	0.57143	0.86061
CT	Fold 1	0.783	44	4	3	4	0.91667	0.57143	0.87273
	Fold 2	0.702	43	3	5	4	0.91489	0.37500	0.83636
	Fold 3	0.840	46	4	4	1	0.97872	0.50000	0.90909
	Fold 4	0.830	45	4	3	2	0.95745	0.57143	0.90741
	Fold 5	0.561	44	1	6	3	0.93617	0.14286	0.83333
	mean	0.743	222	16	21	14	0.94078	0.43214	0.87179
